# Determinants of early initiation of breastfeeding in The Gambia: a population-based study using the 2019–2020 demographic and health survey data

**DOI:** 10.1186/s13006-023-00570-4

**Published:** 2023-06-22

**Authors:** Muhammed L Darboe, Angeline Jeyakumar, Salma M. A. Mansour, Shahanara Valawalkar

**Affiliations:** 1 Department of Health Sciences, Savitribai Phule Pune University (SPPU), Pune, India; 2 University of Johannesburg, Johannesburg, South Africa

**Keywords:** Early initiation of breastfeeding, The Gambia, Malnutrition, Demographic Health Survey (DHS)

## Abstract

**Background:**

Early initiation of breastfeeding within the first hour of life prevents neonatal and infant mortality. Sustainable Development Goals (SDGs) Target 3.2 aims to reduce neonatal mortality and under 5 mortality globally. The decline in the early initiation of breastfeeding in The Gambia coincides with deviations from the SDGs, due to poor indicators of child survival. Our work studied the determinants of early initiation of breastfeeding in The Gambia.

**Methods:**

We used the 2019–2020 Gambia Demographic Health Survey (GDHS) conducted across all regions of the country. Since our population of interest was children born two years preceding the study, we only included children less than 24 months of age, living with an eligible respondent. Thus, a weighted sample of 5691 mother-child pairs was applied in the analysis. We reported summary statistics of individuals’ sociodemographic, obstetrics and antenatal, household, and community-level factors. A logistic regression model was used to determine associations between early initiation of breastfeeding and covariates.

**Results:**

The prevalence of early initiation of breastfeeding was 64.3% (*n* = 3659). Mothers who had secondary education or higher educational level had higher odds of early initiation of breastfeeding (AOR 1.22; 95% CI 1.07, 1.40). Regions with rural population notably Lower and Central and Upper River Region had lower odds of early initiation of breastfeeding [Mansakonko (AOR 0.37; 95% CI 0.26, 0.15), Kerewan (AOR 0.26; 95% CI 0.19, 0.36), Kuntaur (AOR 0.39; 95% CI 0.28, 0.54), Janjanbureh (AOR 0.48; 95% CI 0.35, 0.66) and Basse (AOR 0.64; 95%CI 0.49, 0.85)]. Also, women in the high quintile of the wealth index were more likely to initiate breastfeeding early (AOR 1.29; 95% CI 1.06, 1.57). Four or more antenatal care visits did not increase early initiation of breastfeeding.

**Conclusions:**

The results of the analyses demand affirmative action to improve maternal education, reduce poverty and inequality and empower rural communities in The Gambia. The IYCF component in antenatal care needs to be strengthened. Programs and policies on IYCF must resonate to address determinants of timely breastfeeding initiation to chart progress towards the SDG.

## Background

The World Health Organization (WHO) recommends breastfeeding as a cost-effective and sustainable public health strategy for the prevention of diseases and mortality across the lifecycle [1]. Early initiation of breastfeeding, defined as the initiation of breastfeeding within the first hour of birth, prevents neonatal and infant mortality, which remain a serious public health concern in developing countries [2–4]. Failure to initiate early initiation of breastfeeding increases the risk of infant and child mortality multi-fold. Early initiation of breastfeeding encourages colostrum feeding and offers protection against various childhood illnesses [5–12]. Early intiation of breastfeeding promotes maternal and child bonding that nurtures early child development [13]. Appropriate early intiation of breastfeeding ensures continuity of breastfeeding, which addresses the risk of obesity, overweight, and type 2 diabetes [14].

Sustainable Development Goals (SDGs) Target 3.2 aims to reduce neonatal mortality and under 5 mortality (U5MR) globally. Despite the significant reduction of neonatal and U5MR in sub-Saharan Africa by 41% and 59%; respectively from 1990 to 2020, this region has the highest neonatal mortality rate in the world, at 27 deaths per 1,000 live births (LB) and 54% of all under-five deaths occur in this region [15]. Early intiation of breastfeeding among other indicators is key to child survival, more so in developing countries [16].

Despite the well-established importance of timely breastfeeding initiation, the prevalence of initiation in the first hour is low and uneven in the 13 Economic Community of West African States (ECOWAS). It ranges from 17% in Guinea to 62% in Togo [17]. In The Gambia, early intiation of breastfeeding improved from 48% in 2008 to 52% in 2015 [18] and reduced to 46.5% in 2018 [19] and further fell to 36% between 2019 and 20 [20]. Among different African countries, literature evidence suggested initiation inequalities, specifically delayed feeding in male children, maternal education, wealth index, place and mode of delivery, women’s educational status, and media exposure as contributing factors to poor early intiation of breastfeeding practices [21–23].

Along with a decline in the early intiation of breastfeeding in The Gambia, child survival indicators in The Gambia show deviation from the SDG goals; the neonatal and infant mortality rate and the U5MR are 26, 35, and 49 per 1000 LB; respectively [24]. There is a critical need to determine the current underlying factors that influence early intiation of breastfeeding in The Gambia. In this analysis, we used the recent DHS dataset (DHS 2019) to determine the factors and to provide recommendations to improve early intiation of breastfeeding practices in The Gambia.

## Methods

### Data source, sampling and data collection

The data for this study was extracted from the 2019–2020 Gambia Demographic Health Survey (GDHS). The 2019–2020 GDHS is the most recent in the Demographic and Health Survey series in The Gambia. The survey was conducted across all regions of the country, sampling details can be found in the GDHS report [20].

Women between the ages of 15 and 49 years were approached and, a total of 11,865 women were interviewed. Women, who were permanent household residents, as well as visitors of the household, were both included in the survey. Information ranging from basic sociodemographic data to detailed biomedical information was collected for each respondent. Since our population of interest was children born two years preceding the study, we only included children less than 24 months of age, living with an eligible respondent, and we defined early intiation of breastfeeding as per the WHO [25], which resulted in a total weighted sample of mother-child pairs (*n* = 5691).

### Outcome variable

Our outcome variable was early intiation of breastfeeding [25] as the proportion of children born in the last 24 months who were put to the breast within one hour of birth and received colostrum. This was reported by mothers who were interviewed. Those who initiated after the first hour were considered to have late initiation.

### Covariates

The covariates included individual, household, and community-level factors. Individual factors included sociodemographic characteristics, obstetrics and antenatal care factors.

Sociodemographic characteristics included maternal education (“no education”, “primary”, “secondary and higher”), maternal employment status (“working”, and “not working”), partners education (“no education”, “primary”, “secondary or higher”, and “don’t know”), maternal age in years (“15–24 years”, “25–34 years”, “35–49”), marital status (“never married”, “currently married”, “formerly married”), mothers literacy and exposure to mass media i.e. radio and television were equally included.

Obstetric and antenatal care factors included the place of delivery (“home” or “health facility”), and mode of delivery (“caesarean section” or “normal delivery”).

Antenatal care in DHS measured the percent distribution of live births in the two years preceding the survey by the source of antenatal care during pregnancy. DHS also measured the number of antenatal care visits and timing of the first visit, percent distribution of women who had a live birth in the two years preceding the survey by the number of antenatal care (ANC) visits, and by the timing of the first visit, the median numbers of antenatal care visits, months of pregnancy before first visit, antenatal care visits for live births in the five (three) years preceding the survey, and months of pregnancy at the time of the first visit. For our analysis we categorized the number of antenatal clinic visits as “less than 4 visits”, “4 or more visits”. Mother’s body mass index (BMI) categorized as per the WHO classification. The body mass index (BMI) in DHS is expressed as the ratio of weight in kilograms to the square of height in meters (kg/m2) for adults aged 20–49. A BMI of 18.5–24.9 was considered normal, while BMI of < 18.5 (under-nourished) and ≥ 25 was considered overweight. Categories of birth interval (< 2 years or > 2 years) were considered as per the DHS.

Household-level factors included the wealth index of the household as reported by the GDHS [20]. The wealth index in DHS was assessed using a composite measure of the household’s cumulative living standard using proxy indicators such as ownership of televisions and bicycles; type of housing; and access to water and sanitation facilities. Community-level factors included the place of residence denoted as either “urban” or “rural” and region which included one of the eight administrative regions namely Banjul, Kanifing, Brikama, Mansakonko, Kerewan, Kuntaur, Janjangbureh and Basse.

### Statistical analysis

Weighted samples were applied to allow random selection within the DHS data to minimize any error that could have occurred due to sampling bias. We reported summary statistics of individuals’ sociodemographic, obstetrics, and antenatal care factors, household factors, and community-level factors. We used a binary logistic regression model to determine associations between early intiation of breastfeeding and covariates. The parameters of the model were estimated through a generalized estimating equation approach. The full model was run with those variables that showed *P* < 0.25 in the unadjusted analysis. The final model was reduced using a stepwise approach and only those variables with a *P* – value ≤ 0.05 were included in the final model. Data were analyzed using the statistical software STATA (Version 15.1), odds ratio and 95% confidence interval for both the unadjusted and adjusted model were reported.

**Fig. 1:**
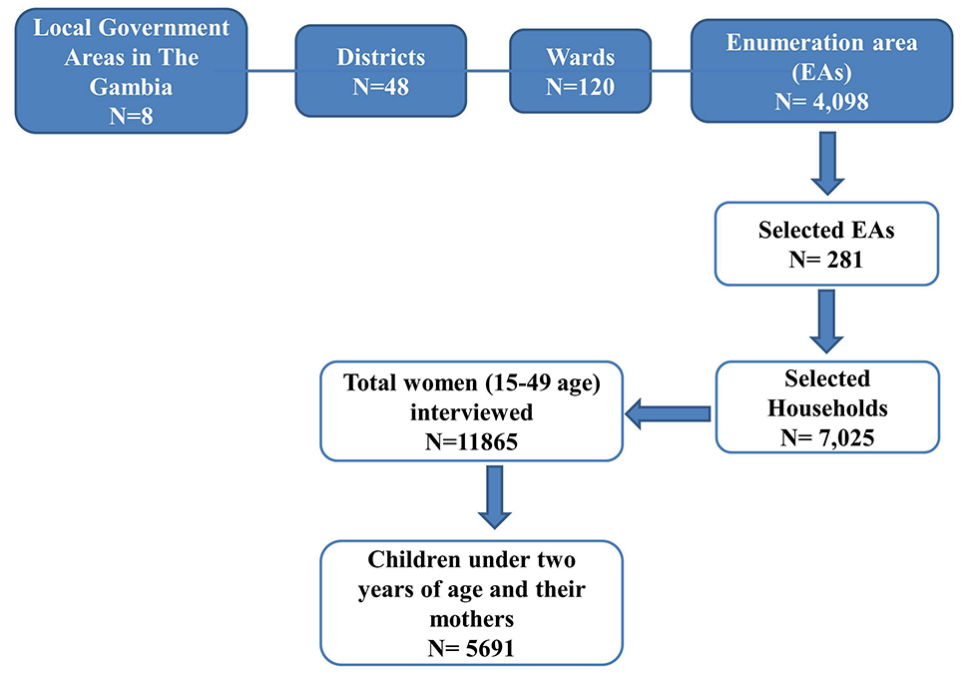
Sample selection from Demographic and Health Survey 2019-20, The Gambia

## Results

### Baseline characteristics

The prevalence of early intiation of breastfeeding within one hour in The Gambia was found to be 64.3% (*n* = 3659). As summarized in Table 1 and 46.6% of children were in urban areas, and over 50% of mothers had no formal education. In addition, 74.6% of mothers had poor literacy skills and could not read or write a sentence. Maternal employment status at the time of the survey was found to be 54%. With regards to maternal age, 24.2% of respondents were between ages 15–24 years while 48.5% were between ages 25–35 years. The majority of respondents (98.5%) were married at the time of the survey.

**Table 1:** Individual, household and community level characteristics of children < 24months of age, The Gambia 2019–2020

Characteristic	Overall (*n* = 5691)	Initiation of breastfeeding	
Individual-level factors	n (%)	Within first hour (n = 3659) n (%)	After first hour (n = 2032) n (%)
**Sociodemographic factors**			
Maternal education			
No education	2975 (53.3)	1976 (66.4)	999 (33.6)
Primary	1032 (18.1)	646 (62.6)	386 (37.4)
Secondary and above	1684 (29.6)	1037 (61.6)	647 (38.4)
Maternal working status			
Working	3079 (54.1)	1981 (64.3)	1098 (35.7)
Not working	2612 (45.9)	1678 (64.2)	934 (35.8)
Partner’s education			
No education	3098 (58.2)	2042 (65.9)	1056 (34.1)
Primary	298 (5.6)	188 (63.1)	110 (36.9)
Secondary	1170 (22.0)	766 (65.5)	404 (34.5)
Higher	335 (6.3)	213 (61.1)	122 (38.9)
Don’t know	419 (7.9)	231 (64.5)	188 (35.5)
Maternal age (years)			
15–24	1378 (24.2)	837 (60.7)	541 (39.3)
25–34	2757 (48.5)	1811 (65.7)	946 (34.4)
35–49	1556 (27.3)	1011 (65.0)	545 (35.0)
Marital status			
Never married	198 (3.5)	110 (55.6)	88 (44.4)
Currently married	5320 (93.5)	3440 (64.7)	1880 (35.3)
Formerly married	173 (3.0)	109 (63.0)	64 (37.0)
Mothers literacy			
Can read part/whole sentences	1446 (25.4)	924 (63.9)	522 (36.1)
Cannot read	4245 (74.6)	2735 (64.4)	1510 (35.6)
Mother listens to radio			
Yes	4294 (75.5)	2793 (65.0)	1501 (35.0)
No	1397 (24.5)	866 (62.0)	531 (38.0)
Mother watches television			
Yes	3975 (69.9)	2546 (64.1)	1429 (35.9)
No	1716 (30.1)	1113 (64.9)	603 (35.1)
**Obstetrics & antenatal care factors**			
Place of delivery			
Home	1058 (18.6)	674 (29.0)	1648 (71.0)
Health facility	4633 (81.4)	2985 (88.6)	384 (11.4)
Mode of delivery			
Caesarean	192 (3.4)	40 (20.8)	152 (79.2)
Non-caesarean	5497 (96.6)	3619 (65.8)	1878 (34.2)
Antenatal care visits			
Less than 4 visit	1064 (19.0)	650 (61.1)	414 (38.9)
4 or more visits	4551 (81.0)	2970 (65.3)	1581 (34.7)
Mothers BMI (kg/m^2^)			
< 18.5	300 (10.3)	204 (68.0)	96 (32.0)
18.5–24.9	1590 (54.7)	1042 (65.5)	548 (34.5)
≥ 25	1015 (35.0)	627 (61.8)	388 (38.2)
**Household factors**			
Wealth Index			
Poorest	1930 (33.9)	1293 (67.0)	637 (33.0)
Poorer	1218 (21.4)	787 (64.6)	431 (35.4)
Middle	1106 (19.4)	691 (62.5)	415 (37.5)
Richer	824 (14.5)	536 (65.0)	288 (35.0)
Richest	613 (10.8)	352 (57.4)	261 (37.5)
**Community level factors**			
Type of residence			
Urban	2651 (46.6)	1565 (59.0)	1086 (41.0)
Rural	3040 (53.4)	2094 (68.9)	946 (37.5)
Region			
Banjul	325 (5.7)	173 (53.2)	152 (46.8)
Kanifing	587 (10.3)	317 (54.0)	270 (46.0)
Brikama	960 (16.9)	534 (55.6)	426 (44.4)
Mansakonko	524 (9.21)	380 (72.5)	144 (27.5)
Kerewan	740 (13.0)	582 (78.6)	158 (21.4)
Kuntaur	776 (13.7)	547 (70.5)	229 (29.5)
Janjanbureh	263 (12.2)	459 (66.2)	234 (33.8)
Basse	693 (19.0)	667 (61.4)	419 (38.6)

Of the total births, 81.4% took place at health facilities, and a low proportion of deliveries (3.4%) were caesarean sections. With regards to antenatal visits, 81% reported having 4 or more antenatal visits. The majority of respondents (54.7%) had a normal BMI of 18.5–24.9, and 35.0% were found to be overweight.

### Predictors of early intiation of breastfeeding

During the 24 months preceding the study, a total of 5691 children were born, of this, 64% were breastfed within the first hour of birth in The Gambia. To determine associations between our dependent variable and covariates, Table 2 shows the unadjusted and adjusted odds ratio.

**Table 2:** Unadjusted and adjusted odds ratio for early initiation of breastfeeding in The Gambia 2019–2020

Characteristic	Unadjusted odds ratio (95% CI)	*p*-value	Adjusted odds ratio (95% CI)	*p-*value
**Individual-level factors**				
*Maternal education*				
No education	1.00		1.00	
Primary	1.18 (1.02, 1.36)	0.026	1.16 (0.99, 1.35)	0.053
Secondary and above	1.23 (1.08, 1.39)	0.001	1.22 (1.07, 1.40)	0.003**
*Maternal working status*				
Not working	1.00			
Working	0.99 (0.89, 1.11)	0.939	-	
*Ethnic identity*				
Mandinka/Jahanka	1.00		1.00	
Wolof	0.82 (0.68, 0.99)	0.042	0.85 (0.70, 1.02)	0.088
Jola/kakaroninka	1.59 (1.24, 2.05)	0.000	1.54 (1.19. 1.98)	0.001**
Fula/Tukulur/Loroboo	1.23 (1.06, 1.43)	0.006	1.25 (1.07, 1.45)	0.004**
Serere	1.29 (0.89, 1.86)	0.168	1.26 (0.87, 1.82)	0.219
Sarahule	1.04 (0.85, 1.26)	0.693	1.07 (0.88, 1.31)	0.454
Creole/Aku	1.13 (0.32, 3.88)	0.844	1.04 (0.30, 3.58)	0.949
Manjago	0.86 (0.40, 1.16)	0.696	0.79 (0.37, 1.68)	0.547
Bambara	0.68 (0.40, 1.16)	0.161	0.69 (0.40, 1.17)	0.175
Other ethnic minorities	2.24 (1.11, 4.52)	0.024	2.11 (1.04, 4.27)	0.037
Non-Gambian	1.32 (1.10, 1.58)	0.003	1.36 (1.13, 1.64)	0.001***
*Partner’s education*				
No education	1.00		-	
Primary	1.13 (0.88, 1.44)	0.327		
Secondary and above	1.03 (0.91, 1.18)	0.563		
Do not know	1.57 (1.28, 1.93)	0.000		
*Maternal age (years)*				
15–24	1.00		1.00	
25–34	0.80 (0.70, 0.92)	0.002	0.80 (0.70, 0.92)	0.002**
35–49	0.83 (0.71, 0.96)	0.018	0.88 (0.75, 1.02)	0.111
*Marital status*				
Never married	1.00			
Currently married	0.68 (0.51, 0.90)	0.009		
Formerly married	0.73 (0.48, 1.11)	0.146		
*Maternal literacy*				
Can read whole/ part sentences	1.00			
Cannot read at all	0.97 (0.86, 1.10)	0.717		
*Mother listens to a radio*			-	
No	1.00			
Yes	0.87 (0.77, 0.99)	0.039		
*Mother watches television*			-	
No	1.00			
Yes	1.03 (0.92, 1.16)	0.558		
**Obstetrics and antenatal care factors**				
*Place of delivery*				
Home	1.00		-	
Health facility	0.96 (0.84, 1.11)	0.657		
*Mode of delivery*				
Vaginal	1.00		1.00	
Caesarean	7.32 (5.14, 10.41)	0.000	7.16 (5.02, 10.21)	0.000**
*Antenatal care visits*				
Less than 4 visits	1.00		1.00	
4 or more visits	0.83 (0.72, 0.95)	0.011	0.81 (0.70, 0.93)	0.003**
*Child put on mothers chest after birth*				
Yes	1.00		1.00	
No	1.45 (1.29, 1.63)	0.000	1.36 (1.21, 1.53)	0.000***
*Maternal BMI (kg/m* ^*2*^ *)*				
18.5–24.9	1.00		-	
< 18.5	0.89 (0.68, 1.16)	0.409		
≥ 25	1.17 (0.99, 1.38)	0.051		
*Birth interval*				
Less than 2 years	1.00		-	
2 years or more	1.02 (0.86, 1.22)	0.744		
**Community-level factors**				
*Wealth Index*				
Poorest	1.00		1.00	
Poorer	1.11 (0.95, 1.29)	0.169	1.08 (0.92, 1.26)	0.306
Middle	1.21 (1.04, 1.42)	0.012	1.15 (0.98, 1.35)	0.076
Richer	1.09 (0.91, 1.29)	0.322	1.03 (0.87, 1.23)	0.681
Richest	1.50 (1.24, 1.81)	0.000	1.29 (1.06, 1.57)	0.009**
*Type of residence*				
Urban	1.00		1.00	
Rural	0.65 (0.58, 0.72)	0.000	0.79 (0.66, 0.95)	0.015**
*Region*				
Banjul	1.00		1.00	
Kanifing	0.96 (0.73, 1.27)	0.823	0.96 (0.73, 1.26)	0.777
Brikama	0.90 (0.70, 1.16)	0.453	0.83 (0.64, 1.07)	0.168
Mansakonko	0.43 (0.32, 0.57)	0.000	0.37 (0.26, 0.51)	0.000***
Kerewan	0.30 (0.23, 0.40)	0.000	0.26 (0.19, 0.36)	0.000***
Kuntaur	0.47 (0.36, 0.62)	0.000	0.39 (0.28, 0.54)	0.000***
Janjanbureh	0.58 (0.44, 0.75)	0.000	0.48 (0.35, 0.66)	0.000***
Basse	0.71 (0.55, 0.91)	0.008	0.64 (0.49, 0.85)	0.002***

A stepwise model with a dichotomous outcome of (0 = initiation within 1 h, 1 = initiation after 1 h) Note: OR (CI) of 1.00 is the reference category, level of significance * p-value of < 0.05, **p-value of < 0.01, ***p-value of < 0.001

Maternal education was found to be associated with early intiation of breastfeeding, where mothers who had secondary education or higher had a 22% (AOR 1.22; 95% CI 1.07, 1.40) higher odds of early intiation of breastfeeding. With regards to sociodemography, regions considered to have a high rural population notably Lower River Region, Central River Region and Upper River Region were found to have significantly reduced odds of early intiation of breastfeeding i.e. [Mansakonko (AOR 0.37; 95% CI 0.26, 0.15), Kerewan (AOR 0.26; 95% CI 0.19, 0.36), Kuntaur (AOR 0.39; 95% CI 0.28, 0.54), Janjanbureh (AOR 0.48; 95% CI 0.35, 0.66) and Basse (AOR 0.64; 95% CI 0.49, 0.85)]. Equally, mothers from the Jola (AOR 1.54; 95% CI 1.19, 1.98), Fula (AOR 1.25; 95% CI 1.07, 1.45), other ethnic minorities (AOR 2.11; 95% CI 1.04, 4.27) and non-Gambian (AOR 1.36; 95% CI 1.13, 1.64) had significantly higher odds of early intiation of breastfeeding compared to the ethnic majority, Mandinka/Jahanka. The wealth index equally showed associations with the early intiation of breastfeeding, where mothers in the highest quintile had 29% higher odds of timely initiation (AOR 1.29; 95% C 1.06, 1.57). Mothers who had four or more visits were less likely to initiate breastfeeding within the first hour compared to those with less than 4 visits (AOR 0.81; 95% CI 0.70, 0.93).

## Discussion

Our work identified a 64% prevalence of early intiation of breastfeeding in The Gambia. Higher education of mothers, rural settings, and a higher wealth index emerged as the major determinants in the DHS 2019–2020 data. The prevalence in 2013 was 53% and the improvement is slow after almost a decade, a pace unfavourable to meet the SDG goals. These and other improvements are the result of an improved scorecard for IYCF as observed in specific indicators such as National Policy, Programme and Coordination, Maternity Protection and Health and Nutrition Care Systems, to name a few [18].

A pooled analysis of the population-based data from 35 sub-Saharan African countries suggests the highest prevalence in Burundi (85.0%), Rwanda (80.5%), and Ethiopia (73.3%) [16]. Regional studies from early 2000 [26] are comparable to our findings depicting little or no change despite the global efforts to improve IYCF practices.

Maternal education is key to child survival, as it disrupts poverty and supports the nutritional gains of the family. In this analysis, among the determinants, maternal education above the secondary level significantly increased the odds of early intiation of breastfeeding. While existing literature suggests cesarean section as the mode of delivery to delay initiation [27, 28], and placing the child on the chest promotes early intiation of breastfeeding [29], our findings are contradictory as documented in other studies [30] that need to be further explored.

Ethnic minorities and non-Gambians showed higher odds of early intiation of breastfeeding. In contrast, the Central, Lower, and Upper River regions showed lower odds of early intiation of breastfeeding in our analyses. Culture and geographical settings significantly influence early intiation of breastfeeding. Despite development in The Gambia, the rural regions in the above geographical settings depend on agriculture as their major source of income, have lower education, less awareness of IYCF practices, and experience multiple pregnancies, coupled with poverty. In rural settings gender disparity in feeding practices, lack of media exposure and awareness, prelacteal feeding practices, misconceptions, and traditional beliefs about discarding colostrum are unrelenting factors that interfere with early intiation of breastfeeding [31–33]. Giving birth at home observed in rural settings increased the risk of prelacteal feeding [34]. Similarities in findings were observed in studies from India, Sri Lanka, and Bangladesh on culture, ethnicity/settings, and beliefs. However, findings on economic status and early intiation of breastfeeding were contradictory. Delayed initiation was higher among wealthier households in these regions [35–37]. Interventions that combine modern and traditional knowledge that is country-specific appear promising [26].

The uniqueness of our work is the selection of only one of the WHO indicators for the assessment of IYCF practices. We studied early intiation of breastfeeding for the following reasons: Literature suggests the determinants of most of the IYCF indicators are similar. However, an indicator’s importance could probably be diluted or generalized when studied together. A first right step “early initiation of breastfeeding” could address several other factors and our work adds to the knowledge of this specific IYCF indicator of The Gambia using a nationally representative sample.

Our work had the following limitations despite its strengths. A secondary analysis of a cross-sectional survey, that shows positive associations do not necessarily reflect causality. Further studies to identify cause-effect relationships are warranted. The results of our study have to be carefully interpreted as education was tested with early intiation of breastfeeding and the awareness or knowledge of early intiation of breastfeeding was not. Also, the number of ANC visits measured in the DHS survey does not reflect the quality of ANC care that could influence early intiation of breastfeeding.

## Conclusions

The results of the analyses demand affirmative action to improve maternal education, reduce poverty and inequality and empower rural communities in The Gambia. The IYCF component in antenatal care needs to be strengthened. Programs and policies on IYCF must therefore resonate to address nutritional and non-nutritional determinants of early initiation of breastfeeding and to chart progress towards the achievement of Sustainable Development Goals.

## Data Availability

Datasets used for this paper are available in the public domain at DHS program website.
